# Alpha- and gammaherpesviruses in stranded striped dolphins (*Stenella coeruleoalba*) from Spain: first molecular detection of gammaherpesvirus infection in central nervous system of odontocetes

**DOI:** 10.1186/s12917-020-02511-3

**Published:** 2020-08-12

**Authors:** Ignacio Vargas-Castro, José Luis Crespo-Picazo, Belén Rivera-Arroyo, Rocío Sánchez, Vicente Marco-Cabedo, María Ángeles Jiménez-Martínez, Manena Fayos, Ángel Serdio, Daniel García-Párraga, José Manuel Sánchez-Vizcaíno

**Affiliations:** 1 VISAVET Center and Animal Health Department, Veterinary School, Complutense University of Madrid, 28040 Madrid, Spain; 2 Fundación Oceanogràfic de la Comunidad Valenciana, 46005 Valencia, Spain; 3 Department of Animal Medicine and Surgery, Veterinary Faculty, Complutense University of Madrid, 28040 Madrid, Spain; 4 Centro de Recuperación de Fauna Silvestre de Cantabria, 39690 Santander, Spain; 5 Tragsatec, 39005 Santander, Spain; 6 Dirección General de Biodiversidad, Medio Ambiente y Cambio Climático, 39011 Santander, Spain

**Keywords:** Herpesvirus, Gammaherpesvirus, Cetaceans, Mass stranding, Striped dolphin, Central nervous system, Cetacean morbillivirus, Cantabrian Sea

## Abstract

**Background:**

Herpesvirus infections in cetaceans have always been attributed to the *Alphaherpesvirinae* and *Gammaherpesvirinae* subfamilies. To date, gammaherpesviruses have not been reported in the central nervous system of odontocetes.

**Case presentation:**

A mass stranding of 14 striped dolphins (*Stenella coeruleoalba*) occurred in Cantabria (Spain) on 18th May 2019. Tissue samples were collected and tested for herpesvirus using nested polymerase chain reaction (PCR), and for cetacean morbillivirus using reverse transcription-PCR. Cetacean morbillivirus was not detected in any of the animals, while gammaherpesvirus was detected in nine male and one female dolphins. Three of these males were coinfected by alphaherpesviruses. Alphaherpesvirus sequences were detected in the cerebrum, spinal cord and tracheobronchial lymph node, while gammaherpesvirus sequences were detected in the cerebrum, cerebellum, spinal cord, pharyngeal tonsils, mesenteric lymph node, tracheobronchial lymph node, lung, skin and penile mucosa. Macroscopic and histopathological post-mortem examinations did not unveil the potential cause of the mass stranding event or any evidence of severe infectious disease in the dolphins. The only observed lesions that may be associated with herpesvirus were three cases of balanitis and one penile papilloma.

**Conclusions:**

To the authors’ knowledge, this is the first report of gammaherpesvirus infection in the central nervous system of odontocete cetaceans. This raises new questions for future studies about how gammaherpesviruses reach the central nervous system and how infection manifests clinically.

## Background

Herpesviruses contain a linear, double-stranded DNA genome (125–290 kpb) within an icosahedral capsid, surrounded by a protein matrix tegument and a lipid bilayer with membrane-associated proteins [1]. One of the most characteristic properties of the herpesviruses is that they can establish latent infections in which viral gene expression is highly limited and no viral particles are produced [2, 3].

Herpesvirus belongs to the *Herpesviridae* family, which consists of three subfamilies: *Alpha*-, *Beta*-, and *Gammaherpesvirinae* [4]. Herpesvirus infections have been observed in a wide range of organisms, including mammals, birds, reptiles, fish, amphibians, and bivalves [1]. So far, *Alpha*- and/or *Gammaherpesvirinae* have been identified in eight cetacean families: Delphinidae, Kogiidae, Ziphiidae, Physeteridae, Monodontidae, Phocoenidae, Iniidae (odontocetes), and Balaenopteridae (mysticetes) [5–11]. Cetacean herpesvirus strains are usually classified according to the sequence of a part of a locus of their DNA polymerase (DNApol) gene [12].

Herpesvirus infections in cetaceans have been associated with pathological processes such as systemic infections [6, 13, 14], encephalitis [15–17], genital lesions [5, 9, 18–21], skin lesions [5, 7, 22–25], nephritis [26], as well as immunosuppression [6]. However, infections have also been detected in cetaceans without any lesions or other clinical manifestations [27]. Molecular detection of herpesvirus infection can provide further insights into virus epidemiology and the infection cycle.

In this study, different strains of *Alpha*- and *Gammaherpesvirinae* were identified in 11 striped dolphins (*Stenella coeruleoalba*) in a mass stranding of 14 dolphins in Cantabria (Spain). Using molecular techniques, gammaherpesvirus were detected in ten dolphins (71.4%, 10/14), and in four of them (28.5%, 4/14) the central nervous system was infected. Alphaherperpesvirus were also detected in four striped dolphins (28.5%, 4/14). To our knowledge, this study is the first to detect gammaherpesvirus in the central nervous system of odontocetes.

## Case presentation

On 18th May 2019, 14 striped dolphins were found stranded in Oyambre Beach, Cantabria, Spain (43°23’34” N, 4°20’03” W). Ten dolphins were found dead, three died during the rescue intervention, and one was euthanized by veterinary staff for humanitarian reasons. The dolphin carcasses were kept refrigerated for up to four days until necropsies could be performed. Detailed necropsies of all animals were carried out as described [28, 29].

Dorsoventral pectoral x-rays were taken in left flipper of all individuals using a conventional veterinary x-ray equipment (Dual Vet X-PL Kit 8Kw 125Kvp 100 mA 250mAs Portable n/s PKL12345) and 70kVp and 6.4mAs exposition setting following Barratclough et al. 2019 [30]. Physiologic maturation was classified based on bone epiphyseal maturation at the distal epiphysis of the radius and ulna [31] as well as total length and gonadal development stage. Twelve out of fourteen individuals showed complete epiphyseal closure in distal radius and ulna, indicating sexual maturity (4 females and 8 males). In the two remaining individuals (1 female and 1 male), distal epiphysis presented no complete closure what has been described in immature striped dolphins.

**Fig. 1:**
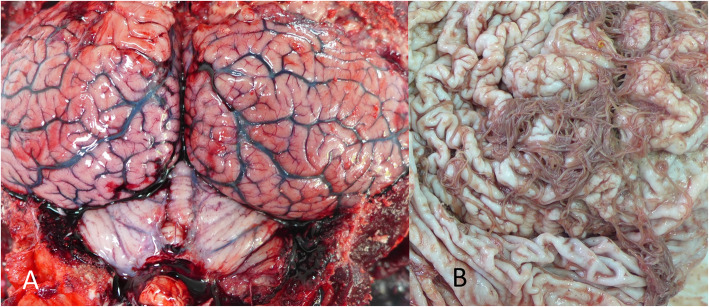
Most relevant macroscopic findings of the stranded striped dolphins. **a.** Cerebrum and cerebellum severe congestion. **b. **Presence of gastric ascarids consistent with *Anisakis* spp

**Fig. 2:**
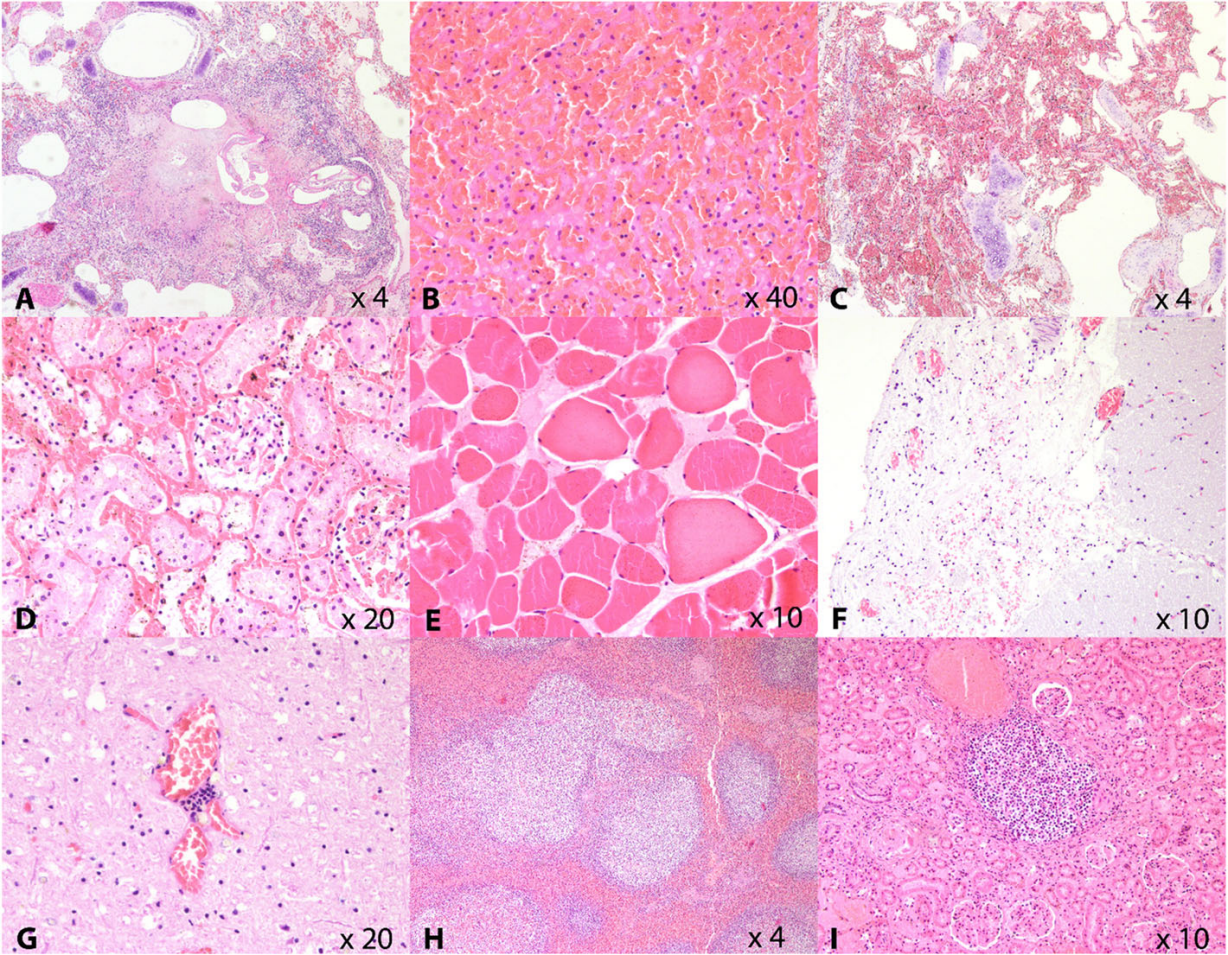
Most relevant microscopic findings of the stranded striped dolphins. **a.** x4PF Lung. Focally extensive granulomatous infiltrate with central necrosis and intralesional sections of a metastrongyle, consistent with Halocercus sp. **b. **x40PF. Liver. Dissociated hepatocytes and hepatic cords, surrounded by extravasated erythrocytes. **c.**x4PF Lung. Severe alveolar interstitial congestion and hemorrhage. **d. **x20PF Kidney. Area of interstitial hemorrhage and associated tubulonecrosis, in area of acute infarction. **e. **x10PF Skeletal muscle. Multifocal monophasic degeneration and myonecrosis. **f.**x10HP Cerebral meninges. Hemorrhage and congestion, and mild lymphoplasmacytic meningitis. **g. **x20HP Cerebrum. Focal lymphoplasmacytic perivascular cuff. **h. **4PF Spleen. Markedly reactive follicles with expanded and depleted germinal centers. **i. **x10PF Kidney. Focal, granulomatous interstitial nephritis

No external injuries or marks caused by interactions with fisheries were observed. Skin lesions were reported in three animals (individuals 8, 9 and 11) and balanitis was reported also in three animals (individuals 6, 7, 17). However, the most common lesion observed during the necropsies was moderate to severe congestion of the cerebrum and cerebellum (Fig. 1a). Body condition [28, 32] was good in all animals (4/5). No significant macroscopic lesions were identified in the thoracic or abdominal cavities. No gastric contents were observed. The internal parasites found, including gastric ascarids (*Anisakis* spp.) (Fig. 1b), pulmonary metastrongyles (*Halocercus* spp.) (Fig. 2a), and hepatic duct trematodes, did not appear to be associated with any severe lesion.

During the necropsies, the following tissues were sampled: skin, blubber, muscle, rete mirabile, pharyngeal tonsils, thyroid, thymus, lymph nodes (tracheobronchial, pre-scapular, and mesenteric), lung, heart, stomach, intestine, liver, pancreas, spleen, adrenal gland, kidney, urinary bladder, ovary/testicle, uterus, penis, mammary gland, cerebrum, cerebellum, and spinal cord. From one of the pregnant females umbilical cord and placenta samples were also collected and from both amniotic fluid and foetus blood. Two sets of tissue samples were collected from each dolphin: the first set was preserved in 10% neutral buffered formalin and used for conventional histopathology, while the second set was stored at − 80 °C and used for virology analysis.

The histopathological findings are summarized in Table 1; Fig. 2. Severe systemic congestion was a common finding, together with diapedesis, microthrombi and associated haemorrhage (Fig. 2b, c, d and f), indicating shock. Acute ischemic tubulonephrosis was observed in some cases associated with infarcts and haemorrhage. (Fig. 2d). Areas of acute monophasic myonecrosis suggestive of exertional rhabdomyolysis were observed in two animals (Fig. 2e). Changes such as myoglobinuria or acute tubulonephrosis frequently associated with exertional rhabdomyolysis were not evident, but may have been masked due to mild autolysis.

**Table 1:** Histological and molecular findings of herpesvirus infection in 14 stranded dolphins

ID	Sex	Main histopathological findings	Tissues tested for HV
6	M	Systemic congestion, severe; Lymphoplasmacytic and suppurative balanitis, mild; Granulomatous and eosinophilic lymphadenitis, mild; Lymphoplasmacytic cholangitis, mild	Cerebellum, spinal cord (A, MN699008), lung, liver, spleen, kidney, skin, pharyngeal tonsils, tracheobronchial lymph node, pre-scapular lymph node, mesenteric lymph node, penis (G, MN698989)
7	M	Systemic congestion, severe; Meningeal hemorrhage and congestion; Lymphoplasmacytic encephalitis, minimal; Lymphoplasmacytic and suppurative balanitis, mild; Lymphoplasmacytic cholangitis, moderate; Polysaccharide myocyte inclusions	Cerebrum (A, MN699009), cerebellum, spinal cord, lung, liver, spleen, kidney, skin, pharyngeal tonsils, tracheobronchial lymph node, pre-scapular lymph node, mesenteric lymph node, penis (G, MN698990)
8	F^a^	Systemic congestion, severe; Lymphoplasmacytic placentitis, minimal; Granulomatous cholangiohepatitis with intralesional trematodes, moderate; Granulomatous and eosinophilic lymphadenitis, moderate; Poxvirus-like dermatitis, mild	Cerebrum, cerebellum, spinal cord, lung, liver, spleen, kidney, skin, pharyngeal tonsils, tracheobronchial lymph node (MT770752), pre-scapular lymph node, mesenteric lymph node.
9	F	Lymphoplasmacytic encephalitis, mild; Systemic congestion and microhemorrhages, severe; suppurative endometritis, mild; Granulomatous and eosinophilic lymphadentitis, moderate	Cerebrum, cerebellum, spinal cord, lung, liver, spleen, kidney, skin, pharyngeal tonsils, tracheobronchial lymph node, pre-scapular lymph node, mesenteric lymph node
10	M	Systemic congestion, severe; Necrosuppurative hepatitis, moderate; Lymphoplasmacytic encephalitis, mild; Granulomatous and eosinophilic lymphadenitis, bronchopneumonia, colitis and gastritis, moderate; Polysaccharide myocyte inclusions	Cerebrum, cerebellum (G, MN698991), spinal cord (G, MN698992), lung (MT770744), liver, spleen, kidney, skin (MT770743), pharyngeal tonsils (MT770742), tracheobronchial lymph node (MT770741), pre-scapular lymph node, mesenteric lymph node, penis (G, MN698993)
11	M	Systemic congestion, severe; Lymphoplasmacytic encephalitis, mild; lymphoplasmacytic hepatitis, mild; Granulomatous and eosinophilic lymphadenitis, bronchopneumonia, colitis, mild; Myonecrosis, mild	Cerebrum, cerebellum, spinal cord (A, MN699007), lung, liver, spleen, kidney, skin, pharyngeal tonsils (MT770748), tracheobronchial lymph node, pre-scapular lymph node, mesenteric lymph node, penis (G, MN698994)
12	M	Systemic congestion, severe; Lymphoplasmacytic encephalitis, mild; lymphoplasmacytic hepatitis, mild; Granulomatous and eosinophilic lymphadenitis, bronchopneumonia	Cerebrum, cerebellum, spinal cord, lung, liver, spleen, kidney, skin (MT770749), pharyngeal tonsils (MT770751), tracheobronchial lymph node (MT770750), pre-scapular lymph node, mesenteric lymph node, penis
13	M	Necrosuppurative hepatitis, moderate; Lymphoplasmacytic encephalitis, mild; Granulomatous and eosinophilic lymphadenitis as well as systemic congestion, severe	Cerebrum (G, MN698995), cerebellum (G, MN698996), spinal cord (G, MN698997), lung, liver, spleen, kidney, skin, pharyngeal tonsils (MT770746), tracheobronchial lymph node, pre-scapular lymph node, mesenteric lymph node (MT770747), penis (G, MN698998)
14	M	Systemic congestion, severe; Lymphoplasmacytic and histiocytic bronchopneumonia, moderate; Lymphoplasmacytic encephalitis, mild; Granulomatous and eosinophilic lymphadenitis, gastritis and duodenitis with intralesional trematodes, moderate	Cerebrum, cerebellum, spinal cord, lung, liver, spleen, kidney, skin (MT770740), pharyngeal tonsils, tracheobronchial lymph node, pre-scapular lymph node, mesenteric lymph node, penis (G, MN698999)
15	F	Systemic congestion, severe; Acute myonecrosis, severe; Granulomatous and eosinophilic lymphadenitis, pneumonia and colitis, mild; Lymphoplasmacytic encephalitis, mild; Lymphoplasmacytic cholangiohepatitis, mild	Cerebrum, cerebellum, spinal cord, lung, liver, spleen, kidney, skin (MT770745), pharyngeal tonsils, tracheobronchial lymph node, pre-scapular lymph node, mesenteric lymph node
16	F^a^	Systemic congestion, severe; Pulmonary haemorrhage and atelectasis, severe; Granulomatous and eosinophilic lymphadenitis and gastritis	Cerebrum, cerebellum, spinal cord, lung, liver, spleen, kidney, skin, pharyngeal tonsils, tracheobronchial lymph node, pre-scapular lymph node, mesenteric lymph node
17	M	Systemic congestion, severe; Granulomatous and eosinophilic lymphadenitis, gastritis, colitis; Penile papilloma; Lymphoplasmacytic balanitis, mild	Cerebrum (G, MN699000), cerebellum (G, MN699001), spinal cord (G, MN699002), lung, liver, spleen, kidney, skin, pharyngeal tonsils, tracheobronchial lymph node, pre-scapular lymph node, mesenteric lymph node, penis (G, MN699003)
18	F	Systemic congestion, severe; Bacterial sepsis; Necrosuppurative hepatitis, moderate; Suppurative and haemorrhagic pneumonia, moderate; Acute myonecrosis, mild; Granulomatous and eosinophilic lymphadenitis and gastritis, mild	Cerebrum, cerebellum, spinal cord, lung, liver, spleen, kidney, skin, pharyngeal tonsils, tracheobronchial lymph node, pre-scapular lymph node, mesenteric lymph node,
19	M	Systemic congestion, severe; Lymphoplasmacytic and histiocytic meningitis, mild; Granulomatous and eosinophilic lymphadentitis and colitis	Cerebrum (G, MN699004), cerebellum, spinal cord (G, MN699005), lung, liver, spleen, kidney, skin, pharyngeal tonsils, tracheobronchial lymph node, pre-scapular lymph node, mesenteric lymph node, penis (G, MN699006)

Samples positive by nested PCR are underlined. Accession numbers of herpesvirus DNA sequences are indicated in parentheses

*Abbreviations*: *A *Alphaherpesvirus, *F* female, *F*^*a*^pregnant female, *G *Gammaherpesvirus, *M *male

Minimal lymphoplasmacytic nonspecific encephalitis was observed in nine animals, while mild lymphoplasmacytic and histiocytic meningitis was detected in one dolphin (Fig. 2f and g). Protozoal cysts consistent with *Toxoplasma* spp. were not observed in any of the animals. Tissue sections from most animals showed minimal inflammation, which was not associated with visible infectious agents or notable tissue damage.

Most lymphoid organs were reactive (Fig. 2h), and the tracheobronchial and mesenteric lymph nodes had mild granulomatous and eosinophilic inflammation, consistent with parasitic infections. Additionally, the lungs (6 individuals), stomach (5 individuals), hepatic bile ducts (4 individuals), and, rarely kidneys (Fig. 2i), showed inflammation and occasional intralesional parasites.

During the virology analysis, tissue samples were evaluated for the presence of herpesvirus and cetacean morbillivirus (CeMV). Standard precautions were taken during all laboratory procedures in order to avoid cross-contamination of samples. All samples were diluted 1:10 with phosphate-buffered saline (PBS) and homogenized using stainless steel 4.8-mm beads (Next Advance, New York, USA). DNA from the homogenates was extracted using the High Pure Template Preparation Kit (Roche Diagnostics, Mannheim, Germany), and total RNA was extracted using the High Pure Viral Nucleic Acid Kit (Roche Diagnostics), based on the manufacturer’s instructions.

A molecular diagnosis of the herpesvirus was carried out based on a previously described pan-herpesvirus nested PCR targeting the DNApol gene [33]. This assay was applied to DNA extracts from the central nervous system (cerebrum, cerebellum, and spinal cord), lung, liver, spleen, kidney, skin and skin lesions, pharyngeal tonsils, tracheobronchial, pre-scapular and mesenteric lymph nodes, and the penis, since these organs are typically targeted by herpesvirus in cetaceans. Ultrapure water was used as negative control, while positive reactions were conducted using known herpesvirus DNA obtained from a harbour porpoise (*Phocoena phocoena*) cerebrum. The agarose bands were purified using the QIAquick® Gel Extraction Kit (Qiagen, Hilden, Germany), and 212-bp amplicons were sequenced completely by Sanger sequencing. The results of the molecular analysis and the herpesvirus sequence accession numbers are provided in Table 1.

DNA sequencing confirmed the presence of herpesvirus in 11 of the 14 striped dolphins (78.57%). Eight novel sequences were identified and deposited in GenBank under accession numbers MN698989 (penis, individual 6), MN698992 (spinal cord, individual 10), MN698995 (cerebrum, individual 13), MN699002 (spinal cord, individual 17), MN699008 (spinal cord, individual 6), MN699009 (cerebrum, individual 7) and MT770740 (skin, individual 6). Sequences with accession numbers MN698990 (penis, individual 7), MN698991 (cerebellum, individual 10), MN698993 (penis, individual 10), MN698994 (penis, individual 11), MN698996 (cerebellum, individual 13), MN698997 (spinal cord, individual 13), MN698998 (penis, individual 13), MN698999 (penis, individual 14), MN69900 (cerebrum, individual, 17), MN699001 (cerebellum, individual 17), MN699003 (penis, individual 17), MN699004 (cerebrum, individual 19), MN699005 (spinal cord, individual 19) and MN699006 (penis, individual 19) were identical to MN698995. Sequence with accession number MN699007 (spinal cord, individual 11) was identical to the previously described sequence KJ156332 of a herpesvirus from a striped dolphin from Canary Islands [17]. Sequences with accession numbers MT770741 (tracheobronchial lymph node, individual 10), MT770742 (pharyngeal tonsils, individual 10), MT770743 (skin, individual 10), MT770744 (lung, individual 10), MT770745 (skin, individual 10), MT770746 (pharyngeal tonsils, individual 13), MT770747 (mesenteric lymph node, individual 13), MT770748 (pharyngeal tonsils, individual 11), MT770749 (skin, individual 13), MT770750 (tracheobronchial lymph node, individual 12), MT770751 (pharyngeal tonsils, individual 13) and MT770752 (tracheobronchial lymph node, individual 8) were identical to MT770740.

**Fig. 3:**
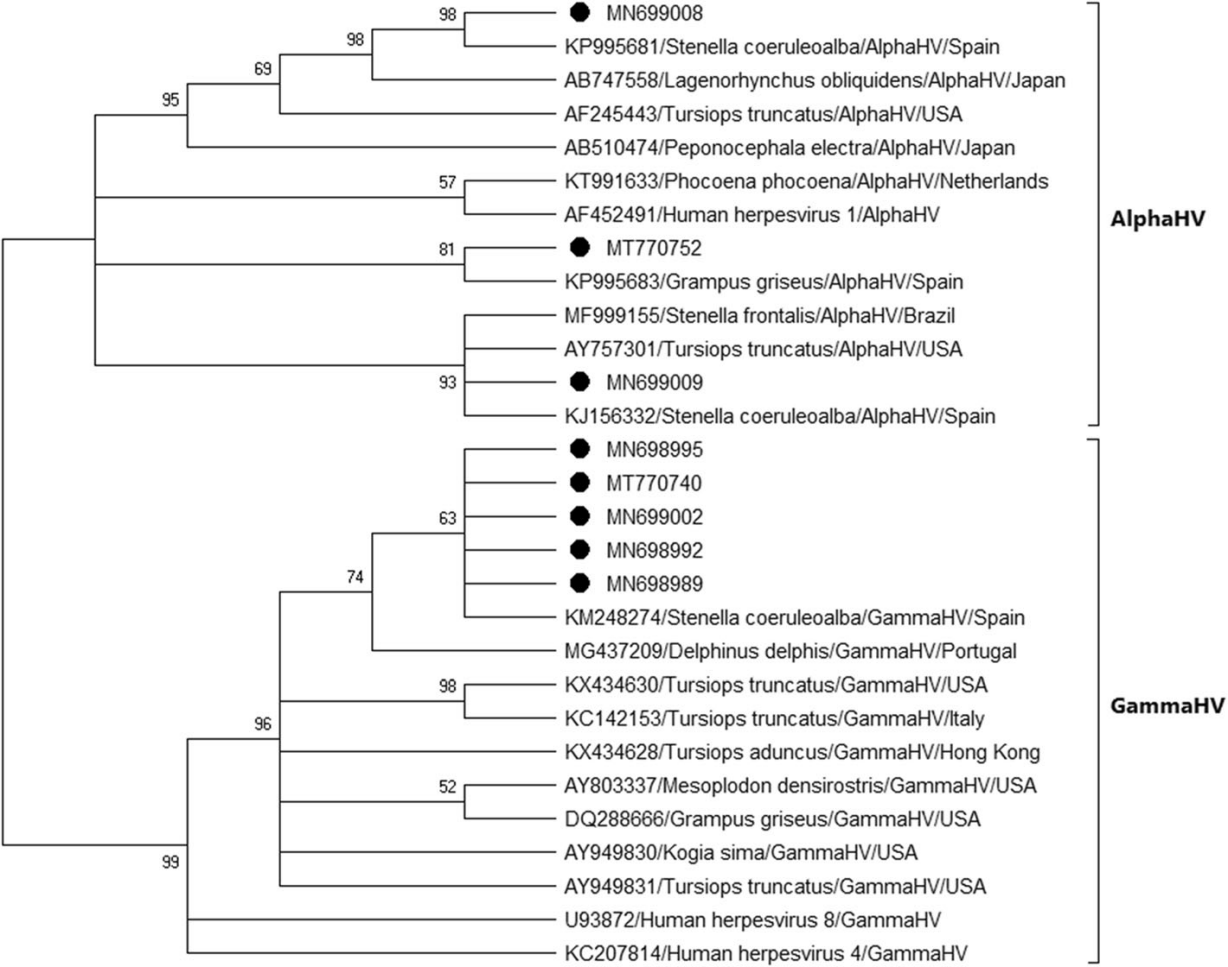
Maximum-likelihood phylogram of herpesviruses, based on partial nucleotide sequence of the DNApol gene. Nucleotide sequences identified in the present study are named only with the accession number. Other cetacean sequences are named according to accession number, host species, subfamily of herpesvirus, and country of origin

**Fig. 4:**
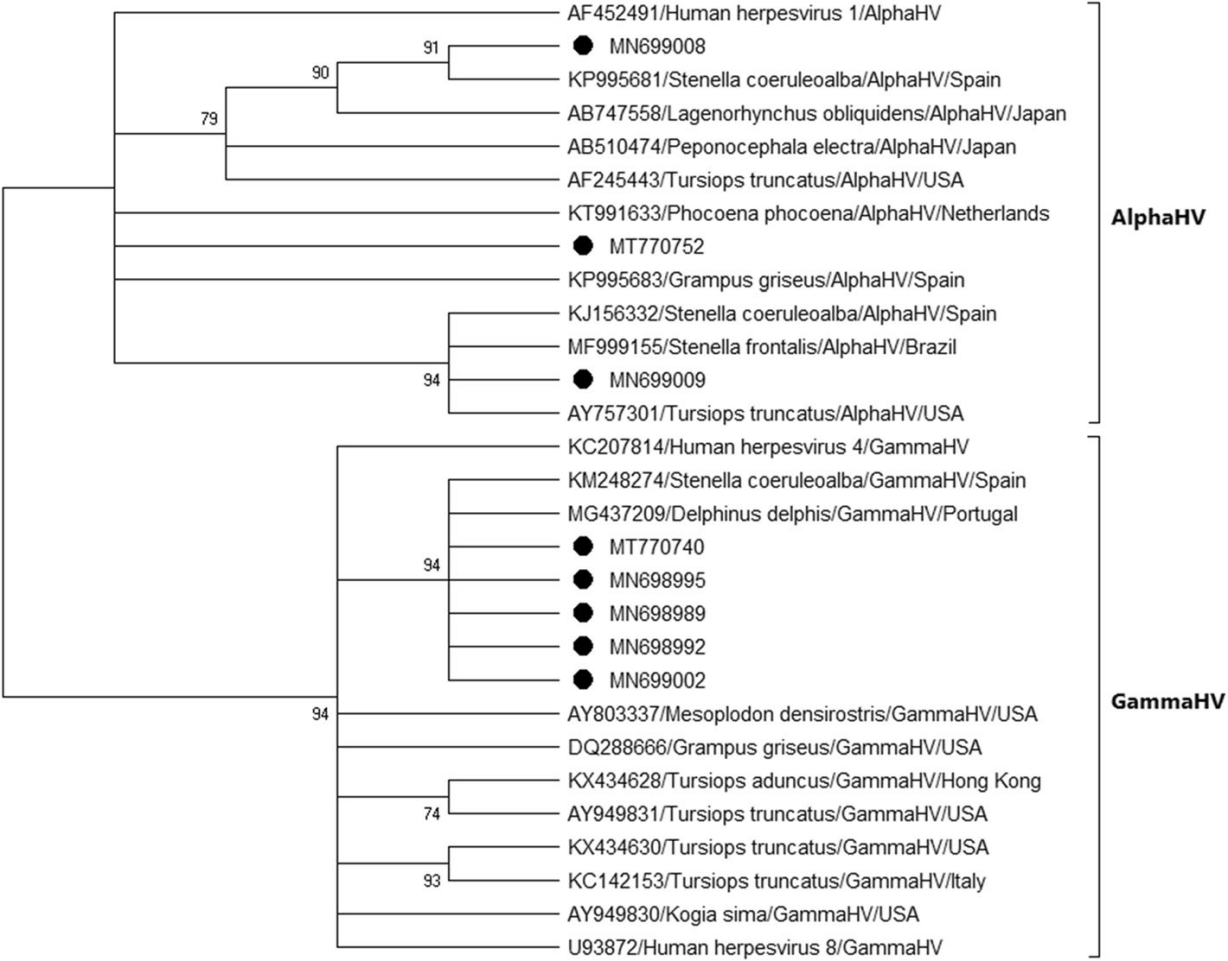
Maximum-likelihood phylogram of herpesviruses, based on partial deduced amino acid sequence of the DNApol gene. Deduced amino acid sequences identified in the present study are named only with the accession number. Other cetacean sequences are named according to accession number, host species, subfamily of herpesvirus, and country of origin

The herpesvirus nucleotide and deduced amino acid sequences were analyzed phylogenetically using the maximum likelihood method in MEGA X software [34] (Figs. 3 and 4). To generate a reliable phylogenetic tree, we confirmed the accuracy of sequence alignments, since the average amino acid p-distance (1-amino acid identity) was 0.44, smaller than the acceptance threshold value (< 0.8) for average p-distance [35, 36]. The reliability of the tree was tested using bootstrap analysis with 1000 replicates. Since only those bootstrap values equal or greater than 70% are considered valid, a consensus tree was computed, accepting the default 50% cut-off value, according to Hall, BG [37] so that some clades are displayed as a politomy (multiple branches coming from one node). Nucleotide and deduced amino acid identities were calculated based on the p-distance.

The phylogenetic analysis revealed that sequences MN699007, MN699008, MN699009 and MT770752 belong to the *Alphaherpesvirinae* subfamily, while sequences MN698989, MN698990, MN698991, MN698992, MN698993, MN698994, MN698995, MN698996, MN698997, MN698998, MN698999, MN699000, MN699001, MN699002, MN699003, MN699004, MN699005, MN699006, MT770740, MT770741, MT770742, MT770743, MT770744, MT770745, MT770746, MT770747, MT770748, MT770749, MT770750 and MT770751 belong to the *Gammaherpesvirinae* subfamily (Figs. 3 and 4). Nucleotide sequences MN698989 to MN699006 and MT770740 to MT770751 clustered with the sequence of a gammaherpesvirus that was found from a genital lesion in a striped dolphin in the Canary Islands, Spain (KM248274) [20]. The nucleotide and deduced amino acid identities for these novel sequences and sequence with accession number KM248274 were: 98.45% and 100%, respectively, for MT770740, MN698995 and MN698989; 97.67% and 97.67%, respectively, for MN699002; and 96.90% and 97.67%, respectively, for MN698992. Nucleotide sequence MN699008 clustered with the sequence of an alphaherpesvirus from a striped dolphin from the Valencian Community coast, Spain (KP995681) (unpublished) (nucleotide identity: 96.97%, deduced amino acid identity: 100.00%). Nucleotide sequence MN699009 was part of a polytomy with alphaherpesviruses obtained from a bottlenose dolphin (*Tursiops truncatus*) from Florida, USA (AY757301) [5] (nucleotide identity: 91.80%, deduced amino acid identity: 95.45%); an Atlantic spotted dolphin (*Stenella frontalis*) from Sao Francisco do Sul, Brazil (MF999155) [7] (nucleotide identity: 95.85%, deduced amino acid identity: 97.50%); and a striped dolphin from Canary Islands (KJ156332) [17] (nucleotide identity: 97.01%, deduced amino acid identity: 100%). Nucleotide sequence MT770752 clustered with the sequence obtained in a Risso’s dolphin (*Grampus griseus*) from a striped dolphin from the Valencian Community coast, Spain (KP995683) (unpublished) (nucleotide identity: 86.09%, deduced amino acid identity: 86.49%).

RNA extracted from tissues targeted by CeMV were assayed for the virus using a reverse transcription-PCR method that targets the fusion protein gene and is based on the Universal Probe Library platform [38]. These tissues were cerebrum, cerebellum, pharyngeal tonsils, lung, tracheobronchial and pre-scapular lymph nodes, as well as kidney. Ultrapure water was used as negative control, while striped dolphin CeMV-positive lung RNA was used as positive control. All samples were negative for CeMV infection.

Additionally, DNA extracts from four skin lesions were tested for Poxvirus by conventional PCR [39]. Ultrapure water was used as negative control, while striped dolphin poxvirus-positive skin DNA was used as positive control. All samples were negative.

## Discussion and conclusions

Histopathology and PCR were used to assess the effect of herpesvirus infections on 14 striped dolphins from the Cantabrian Sea stranded on a beach in Cantabria, Spain. Several cetacean mass stranding causes have been proposed, and include: receding tides, navigational errors, geomagnetic disturbances, disturbance of echolocation, following prey inshore, escaping from predators, disease in one or more individuals in a social group, algal toxins or meteorological events [40]. The cause of this massive stranding event could not be determined. CeMV is a virus that has been associated with cetacean mass mortality events worldwide [41], including in Spain [42–44], but in this mass stranding event all samples analyzed were negative for CeMV. However, molecular analysis identified 34 herpesviral sequences (four alphaherpesviruses and 30 gammaherpesviruses) in 11 out of 14 stranded dolphins, of which eight sequences were novel. This is the first study to detect gammaherpesvirus infection in the central nervous system of an odontocete species.

Gammaherpesvirus has been associated with skin and genital lesions in cetaceans [5, 7, 18–21]. Here, we described three cases of balanitis (dolphins 6, 7 and 17), one of them with an associated papilloma (dolphin 7). Gammaherpesvirus sequences were determined in the molecular analysis of the three cases. However, in this study we also report the detection of gammaherpesvirus sequences in central nervous system (cerebrum, cerebellum and spinal cord) in several striped dolphins. How gammaherpesvirus reaches the central nervous system is unclear. Gammaherpesvirus are lymphotropic [45], therefore, it is most likely that it reaches the central nervous system by blood. Gammaherpesvirus were found also in lymph tissues, such as, pharyngeal tonsils (dolphins 10, 11, 12, 13), tracheobronchial (dolphins 10 and 12) and mesenteric lymph nodes (dolphin 13); and lungs (dolphin 10). These findings would be consistent with possible viremia and subsequent tissue invasion.

However, some neurotropism has also been suggested in human gammaherpesviruses [46–50]. Thus, comparing it to clearly neurotropic viruses, another potential route may be via autonomic neurons in the genitals, similar to the route taken by human herpesviruses type 1 and type 2 to reach the spinal cord [51]. The fact that two individuals (dolphins 17 and 19) had gammaherpesvirus infection restricted to central nervous system and penis could be explained by this potential route. The virus may have migrated from the penis to the spinal cord via ganglia or peripheral nerves, after which it entered the cerebellum and the cerebrum. However, additional research is necessary to study and demonstrate the route of migration to the central nervous system. In addition, further studies will also be necessary to determine which factors allow gammaherpesvirus to reach the central nervous system, since only four out of ten (40%) gammaherpesvirus cases had central nervous system infection. Another subject of study is the possibility that sex acts as a risk factor in this type of infection, since this gammaherpesvirus species was identified in all males, while only one of the five stranded females was positive for this virus. This female presented the infection on the skin. In contrast, in seven of the nine males the infection affected several of the studied organs (from 2 to 7, depending on the individual). Furthermore, the 4 cases of gammaherpesvirus infection in the central nervous system were males. Nevertheless it would be necessary to study a greater number of animals, and both female and male genitalia, in order to elucidate sex as a risk factor.

Another aspect to consider was the lack of lesions caused or potentially associated with a herpesviral infection apart from the three cases of balanitis. Tissue reaction and inflammation associated with the presence of the virus could help elucidate the route or the pathologic relevance of the infection. The minimal lymphoplasmacytic inflammation within the central nervous system in nine of the animals was nonspecific, and could not be associated with any specific agent, and it was not severe enough to trigger clinical manifestations. Findings thus support the hypothesis that virus was causing subclinical infection at the least, at the time of death.

Both subfamilies, *Alphaherpesvirinae* and *Gammaherpesvirinae*, have been detected in skin and genitals from cetaceans [5, 7, 9, 18–25], but only alphaherpesviruses have been identified in the central nervous system of odontocetes. Nevertheless, central nervous system gammaherpesvirus infection was previously described in mysticetes [11]. To our knowledge this is the first report of gammaherpesvirus detection in the central nervous system of dolphins. It has been described that human gammaherpesvirus can be found in healthy human brains [47, 48] and Murine gammaherpesvirus 68 was found to be able to cause a persistent infection in mice central nervous system [46] suggesting a neuroinvasive and neuropersistent potential. This discovery enhances our understanding of herpesvirus infection in cetaceans and brings up many questions that need further investigation about how herpesvirus infection progresses in cetaceans, particularly in the central nervous system, what are the risk factors of such infection and how infection manifests clinically.

## Data Availability

DNA sequences have been deposited in GenBank (accession numbers: MN698989-MN699009 and MT770740-MT770752).

## Abbreviations

CeMV: Cetacean morbillivirus
PCR: Polymerase chain reaction
DNApol: DNA polymerase
